# Exploratory Analysis of TLR2, TLR4, Interleukin 6 and Interleukin 10 Gene Polymorphisms in Relation to Clinical Early-Onset Sepsis in Preterm Neonates: A Single-Center Study

**DOI:** 10.3390/life16010103

**Published:** 2026-01-11

**Authors:** Melinda Baizat, Mihaela Iancu, Gabriela Zaharie, Monica Hășmășanu, Melinda Matyas, Ioana Cristina Rotar, Roxana Liana Lucaciu, Adriana Corina Hangan, Sidonia Gog Bogdan, Lucia Maria Procopciuc

**Affiliations:** 1 Department of Neonatology, Zalău County Emergency Hospital, 450129 Zalău, Romania; baizat.irina@umfcluj.ro; 2 Medical Informatics and Biostatistics, Faculty of Nursing and Health Science, “Iuliu Hațieganu” University of Medicine and Pharmacy, 400012 Cluj-Napoca, Romania; 3 Department of Neonatology I, “Iuliu Hațieganu” University of Medicine and Pharmacy, County Emergency Hospital, 400006 Cluj-Napoca, Romania; gzaharie@umfcluj.ro (G.Z.); monica.hasmasanu@gmail.com (M.H.); melinda.matyas@umfcluj.ro (M.M.); 4 Department of Obstetrics and Gynecology, “Iuliu Hațieganu” University of Medicine and Pharmacy, County Emergency Hospital, 400349 Cluj-Napoca, Romania; cristina.rotar@umfcluj.ro; 5 Department of Pharmaceutical Biochemistry and Clinical Laboratory, Faculty of Pharmacy, “Iuliu Hațieganu” University of Medicine and Pharmacy, 400349 Cluj-Napoca, Romania; liana.lucaciu@umfcluj.ro; 6 Department of Inorganic Chemistry, Faculty of Pharmacy, “Iuliu Hațieganu” University of Medicine and Pharmacy, 400012 Cluj-Napoca, Romania; adriana.hangan@umfcluj.ro; 7 Department of Surgery and ATI, Faculty of Veterinary Medicine, University of Agricultural Sciences and Veterinary Medicine, 400372 Cluj-Napoca, Romania; sidonia.bogdan@usamvcluj.ro; 8 Department of Medical Biochemistry, “Iuliu Hațieganu” University of Medicine and Pharmacy, 400349 Cluj-Napoca, Romania; lprocopciuc@umfcluj.ro

**Keywords:** neonatal sepsis, *TLR2*-*Arg753Gln*, *TLR4*-*Asp299Gly*, *IL6*-*174G/C*, *IL10*-*1082G/A*

## Abstract

(1) Background: Neonatal sepsis continues to be one of the leading causes of mortality and morbidity, particularly in underdeveloped countries. We aimed to compare laboratory parameters between clinical early-onset sepsis (clinEOS) and NNNon-clinEOS groups and to evaluate the association between *TLR2*-*Arg753Gln*, *TLR4*-*Asp299Gly*, *IL6*-*174G/C,* and *IL10*-*1082G/A* gene single-nucleotide polymorphisms and clinical EOS susceptibility in preterm newborns. (2) Materials and Methods: Genotyping of the *TLR2*, *TLR4*, *IL6*, and *IL10* polymorphisms was performed in 36 preterm neonates with polymerase chain reaction (PCR) and restriction fragment length polymorphism analysis (RFLP). Logistic regression analysis was used to test the associations between the studied gene polymorphisms and EOS susceptibility. (3) Results: Statistically significant differences in gestational age and birth weight were observed between the two groups, with preterm neonates with clinical EOS having a lower mean gestational age (mean (SD): 29.4 (2.8) weeks vs. 32.6 (1.1); *p* = 0.00002) and a lower mean birth weight (1342.1 (446.5) gr. Vs. 1984 (376.9)) than preterm neonates without clinical EOS. C-reactive protein (CRP) values measured on the first day significantly increased in the clinEOS group compared with the non-clinEOS group (median, 95% CI: 0.80 [0.40, 1.15] vs. 0.30 [0.02, 0.50]). The mean number of neutrophils significantly decreased in the preterm neonates with clinical EOS (mean difference: 17.3%; 95% CI: [4.0%, 30.5%]; *p* = 0.0126) and non-clinEOS group (mean difference: 20.8%; 95% CI: [1.8%, 39.9%]; *p* = 0.0354) between the first and seventh hospitalization days. In the dominant model, the A/G + A/A variant genotype of the *IL10*-*1082G/A* polymorphism significantly increased the odds of clinical EOS compared with the GG genotype (OR = 5.25; *p* = 0.0322), but the gestational-age-group adjusted model yielded *p* = 0.0752. (4) Conclusions: The results of the current study suggest that *IL10*-*1082G/A* gene polymorphism is a significant risk factor for clinical early-onset sepsis development in preterm neonates, but there was no evidence of a gestational age-group independent direct effect of *IL10*-*1082G/A* gene polymorphism on clinical EOS susceptibility. The results should be considered as exploratory.

## 1. Introduction

Neonatal sepsis continues to be one of the leading causes of mortality and morbidity, particularly in underdeveloped countries [1].

The Global Burden of Disease (GBD) Study 2016/2017 estimated 1.3 (95% CI; 0.8 to 2.3) million annual incident cases of neonatal sepsis worldwide, resulting in 203,000 (95% CI; 178,700 to 267,100) sepsis-attributable deaths [2].

In newborns, protection against sepsis is provided by both innate and acquired immunity. The first line of defense against sepsis is represented by the infant’s own immune system, which includes physical barriers (the skin and mucous membranes of the respiratory and digestive membranes), immune cells (neutrophils, macrophages, and natural killer cells—NK cells), and soluble factors. This type of immunity acts rapidly, is non-specific, and could explain the increased susceptibility to infections. Acquired immunity, in fact, maternal immunity, is realized by IgG antibodies, which are transferred from the mother to the fetus through the placenta. This type of immunity is specific, and offers temporary protection, reflects the mother’s immunity against infections, acts during the first months of life, and provides specific protection against pathogens for 3–6 months. IgG levels gradually decrease after birth until the infant begins to produce its own antibodies [3].

Newborns rely on their innate immune system for anti-infectious defense, which provides a rapid and non-specific response to pathogenic microorganisms they come into contact with [4].

To create comprehensive evidence-based recommendations for the recognition and management of children with septic shock or other sepsis-associated acute organ dysfunction in 2020, the guide Management of Septic Shock and Sepsis-Associated Organ Dysfunction in Children was published [5].

The gold standard in the diagnosis of sepsis remains blood culture analysis, but due to its inherent limitations, such as the time until the result appears and the required amount of blood, it is a difficult goal to achieve in the pediatric population. That is why it is recommended for the early diagnosis and management of cases to use institutional protocols. Adherence to protocols reduces variability in care and improves outcomes for children with septic shock or other organ dysfunction associated with sepsis. These are considered best practice [5].

Sepsis is categorized as early-onset if diagnosed within the first 72 h of life, which is due to perinatal risk factors, or late-onset if diagnosed after 72 h and secondary to nosocomial risk factors [6].

The pathogenesis of neonatal sepsis still requires investigation; the lack of consensus in the definition of neonatal sepsis, even more so in the case of premature newborns, makes it more difficult to understand its pathogenesis [7].

There are known risk factors for the development of sepsis in the newborn, especially in preterm neonates. These are as follows: small gestational age, low birth weight, a positive maternal vaginal culture for group B streptococcus (GBS), prolonged rupture of membranes, maternal intrapartum fever, and male sex. Chorioamnionitis is associated with the highest risk of subsequent occurrence, clinical or culture-proven sepsis [8,9].

Bacteria are the pathogens most frequently involved in the development of sepsis infection by Gram-negative organisms, particularly Pseudomonas species, carries a higher risk for a fulminant course and death than infection by other pathogen groups. Gram-positive causes of sepsis are dominated by GBS and coagulase-negative staphylococci (CoNS) [10].

New data in adults and children demonstrate simultaneous pro-inflammatory/anti-inflammatory responses where the magnitude of either response may determine outcome [11].

The clinical manifestations vary considerably and are non-specific, which makes the diagnosis of early neonatal sepsis difficult and predisposes them to excessive antibiotic use.

In the case of prematurity, the signs and symptoms of neonatal sepsis overlap with comorbidities associated with prematurity, thus making differential diagnosis more difficult [12].

TLRs function as sensors of innate immunity, monitor and identify various pathogens, and become the body’s first barrier against pathogenic microorganisms. Binding to TLRs (toll-like receptors) stimulates a response by releasing cytokines, chemokines, complement proteins, and clotting factors. While newborns and adults have similar TLR expression levels, subsequent responses from PAMP (pathogen-associated molecular patterns)–TLR binding differ in the case of preterm neonates [6,13,14].

Previous studies have shown that during infection, TLR changes in preterm infants are considerably smaller than those in full-term infants, thus leading to diminished leukocyte activation and secretion of pro-inflammatory cytokines, as well as reduced over-regulation of various factors [15,16].

TLR2 is now recognized as a receptor for bacterial lipoproteins and lipoteichoic acids in Gram-positive bacteria. In contrast, TLR4 is identified as the ultimate receptor for lipopolysaccharides (endotoxins) in Gram-negative organisms [17]. A different manifestation of infection in critically ill patients suggests that host genetics influence the magnitude of the innate immune response and the severity of sepsis via the modulation of TLR expression and function [18].

Polymorphisms or mutations in *TLR* genes are associated with increased risk for infection in adults [19] and children [20,21] but are less well characterized in neonates.

Several single-nucleotide polymorphisms (SNPs) in *TLR2* and *TLR4* have been identified in children and adult patients presenting with sepsis and admitted to intensive care units [22,23].

The gene encoding TLR2 is located on the long arm of chromosome 4 (4q32), where several SNP-type polymorphisms (SNPs) have been identified. *Arg753Gln* (rs5743708, *G2258A*) is one of the most studied SNPs found in multiple pathologies. The *TLR2*-*Arg753Gln* variant is caused by an arginine substitution with glutamine at locus 753, which results in a decreased response of macrophages to bacterial peptides [24].

Previous studies have suggested that *TLR2*-*Arg753Gln* may lead to decreased activation of intracellular signaling pathways [25]. Moreover, population studies have shown that *TLR2* polymorphisms could influence the body’s overreaction in several diseases such as cancer, tuberculosis, infective endocarditis, and sepsis [26].

TLR4 is a protein composed of 224 amino acids that is encoded in humans by a gene located on the 9q32–q33 chromosome and contains three exons. Several single-nucleotide polymorphisms (SNPs) have been identified in the *TLR4* gene, some of which are strongly associated with increased susceptibility to Gram-negative bacterial infections and an increased incidence of sepsis [27]. TLR4 activity and function appear to be modulated by this genetic variation. One of these genetic polymorphisms is the point mutation *896A/G* (rs4986790), which determines the substitution of aspartic acid with glycine in position 299 of the protein (*Asp299Gly*). In humans, the frequency of this polymorphism is higher than 5% [28,29]. The *Asp299Gly* polymorphism is located in the leucine-rich repeat (LRR) domain of exon 3, which links to pathogen-associated molecular pattern recognition (PAMP). The *TLR4*-*Asp299Gly* polymorphism has been found to prolong the length of hospitalization in adult patients with urinary tract infections [30].

Interleukin 6 (IL6) plays a significant role in the immune response and regulation of inflammatory reactions. Elevated IL6 levels are associated with an increased risk of severe sepsis and an increased death rate from severe sepsis [31]. The *IL6* gene, located on chromosome 7p21 and spanning 5 kb, contains four introns and five exons. Several polymorphisms have been identified in the promoter region of the IL6 gene. Among these, the common polymorphism *174G/C* (rs1800795) contains a DNA-binding site for the nuclear factor IL6, a transcription factor that can interact with estradiol receptor complexes to regulate IL6 gene expression [32]. The G–C polymorphism at position 174 of the *IL6* gene (rs1800795) influences negative outcomes in several inflammatory diseases, including sepsis [33,34].

Interleukin 10 (IL10) has been identified as one of the key anti-inflammatory cytokines in the inflammatory cascade, as it decreases the production of inflammatory molecules such as TNF-α, interferon (IFN)-γ, interleukin 12 (IL12), reactive nitrogen oxide metabolites, major histocompatibility complex molecules, and inhibits antigen-specific cytotoxic T-cells [35]. IL10 is a potent immunoregulatory cytokine that is widely known for its anti-inflammatory and B-cell-stimulating functions. IL10 is one of the anti-inflammatory cytokines that suppresses the actions of IL6, TNF-α, and interleukin 8 (IL8) [36]. The gene encoding IL10 is located on chromosome 1 (1q31-1q32) [37].

Conversely, excess IL10 induces immunosuppression in sepsis and increases mortality by impairing bacterial clearance in pneumococcal pneumonia [38]. Depending on its chromosomal localization and functional relevance, IL10 is a multifunctional cytokine that can not only inhibit pro-inflammatory cytokine synthesis but also reduce antigen presentation and macrophage activation [39]. The genetic polymorphism *1082G/A*, located in the promoter region of *IL10*, can affect IL10 expression [40]. The *IL10*-*1082G/A* polymorphism is caused by the substitution of guanine with adenine in position 1082 of the gene [41]. The impact of sepsis and multiorgan dysfunction is overwhelming for the patient and for the health system. The total annual average costs of CVD (cardiovascular disease) in adult patients, in Poland (both direct and indirect), amount to 37.5 bn PLN (8.8 bn EUR), and it is 1.89% of GDP. The total estimated indirect costs of CVD amount to 31.3 bn PLN (7.3 bn EUR) and significantly exceed the direct medical costs, which amount to 6.1 bn PLN (1.4 bn EUR) [42,43].

That is why it is important to develop sepsis diagnosis and management protocols that limit the clinical and economic consequences of this complex pathology.

The main objectives of the present study were to test differences in distributions of paraclinical parameters between preterm neonates with clinical early-onset sepsis (clinEOS) and those without clinical early-onset sepsis (Non-clinEOS) and to quantify the effects of *TLR2*, *TLR4*, *IL*6, and *IL*10 gene polymorphisms on clinical early-onset sepsis susceptibility. Secondly, we aimed to test whether the effects of the studied SNPs were independent after adjustment for gestational age group (very preterm versus moderate–late preterm).

## 2. Materials and Methods

### 2.1. The Study Groups

The studied samples included 36 premature newborns who met the selection criteria, admitted to the Clinic of Gynecology I, Cluj-Napoca, a tertiary care center for newborns in Romania, between 2016 and 2017.

Based on the laboratory results and clinical examinations, all preterm neonates were divided into two groups: a clinEOS group, which includes 26 patients with sepsis in the first 72 h of life, and a Non-clinEOS group, which includes 10 patients. Preterm neonates included in the clinEOS group were premature newborns with a GA of <36 + WG (weeks of gestation) without congenital malformations; with at least one of the risk factors for early sepsis, including premature rupture of the amniotic membranes more than 18 h before birth, chorioamnionitis, maternal urinary tract infection, intrapartum maternal fever, meconium amniotic stain liquid, a low Apgar score, and the need for resuscitation in the delivery room; with at least two of the clinical signs and symptoms of neonatal sepsis that start in the first 72 h of life, i.e., respiratory distress, tachy-bradypnea, apnea episodes, the need for invasive or non-invasive respiratory support, cardiac decompensation, tachy-bradycardia, a TCR of greater than 3 s, hypotension, hypo/hyperglycemia, acid–base disorder, early hyperbilirubinemia that started before 24 h of life, convulsions, and lethargy; and patients who received antibiotic treatment in different combinations.

Subjects included in the Non-clinEOS group were preterm newborns without clinical manifestations of sepsis, and they did not receive antibiotic treatment.

All procedures performed in this study involving human participants were in accordance with the ethical guidelines of the Declaration of Helsinki. Participants were enrolled according to established eligibility criteria.

### 2.2. TLR2-Arg753Gln (rs 5743708), TLR4-Asp299Gly (rs 4986790), IL6-174 G/C (rs 1800795), and IL10-1082G/A (rs 1800896)

The *TLR2*-*Arg753Gln* genetic variation is an *MspI* restriction fragment length polymorphism (RFLP). The flanking 129 base pair (bp) region was amplified using primers described by Dhifallah et al. [44]. The *TLR4*-*Asp299Gly* genetic variation is a *NcoI* restriction fragment length polymorphism (RFLP). The flanking 188 bp region was amplified using primers described by Dhifallah et al. [44]. The *IL6*-*174 G/C* genetic variation is a *LweI* restriction fragment length polymorphism (RFLP). The flanking 610 bp region was amplified using primers described by Aker et al. [45]. The *IL10*-*1082 G/A* polymorphism is a *XagI* restriction fragment length polymorphism (RFLP). The flanking 377 bp region was amplified using primers described by Cordeiro et al. [46].

#### 2.2.1. DNA Extraction

Two-milliliter blood samples were collected from patients diagnosed with early neonatal sepsis in tubes containing EDTA as an anticoagulant. High molecular weight DNA was extracted using a Zymoresearch kit (Quick-DNAMiniprep, Kit-Zymo Research Corporation, Freiburg, Germany). The DNA samples were stored at −20 °C until the PCR procedure.

#### 2.2.2. Genotyping Analysis

The *TLR2*, *TLR4*, *IL6*, and *IL10* polymorphism genotyping was performed using polymerase chain reaction (PCR) and restriction fragment length polymorphism analysis (RFLP).

##### PCR

The PCR was carried out using a 20 ng DNA template, 200 mM of deoxynucleotide triphosphates (dNTPs), 0.2 M of forward and reverse primers, 2.0 mM of MgCl_2_, and 0.625 units of *Taq polymerase* in a PCR buffer containing 50 mM KCl and 10 mM Tris-HCl (pH 8.3). The lyophilized primers were obtained from Eurogentec (Kaneka Eurogentec S.A. Biologics Division, Liege, Belgium) and were reconstituted in sterile deionized water (DDW) and stored at −20 °C.

Genomic DNA was amplified with an iCycler C1000 BioRad (Bio-Rad Life Science, Hercules, CA, USA) using the following PCR conditions:

*TLR2-Arg753Gln:* Initial denaturation at 95 °C for 60 s, followed by 34 cycles of denaturation at 95 °C for 10 s, primer annealing at 64.8 °C for 30 s, and a final extension at 72 °C for 30 s. A 129 bp common product was obtained.

*TLR4-Asp299Gly:* Initial denaturation at 95 °C for 60 s, followed by 34 cycles of denaturation at 95 °C for 10 s, primer annealing at 60 °C for 1 min, 20 s, extension at 72 °C for 15 s, and a final extension at 72 °C for 30 s. A 188 bp common product was obtained.

*IL6-174G/C*: Initial denaturation at 95 °C for 60 s, followed by 34 cycles of denaturation at 95 °C for 10 s, primer annealing at 55.3 °C for 20 s, extension at 72 °C for 15 s, and a final extension at 72 °C for 30 s. A 610 bp common product was obtained.

*IL10-1082G/A*: Initial denaturation at 95 °C for 60 s, followed by 34 cycles of denaturation at 95 °C for 10 s, primer annealing at 52.3 °C for 20 s, extension at 72 °C for 30 s, and a final extension at 72 °C for 30 s. A 377 bp common product was obtained.

The specificity of the PCRs was checked using electrophoresis on a 2% agarose gel prepared in 1X TBE buffer containing ethidium bromide (Thermo Fisher Scientific, Waltham, MA, USA) (0.5 µg/mL). Visualization was performed under a UV transilluminator (Thermo Fisher Scientific, Waltham, MA, USA).

##### RFLP Analysis

An amount of 6 µL of amplified DNA was digested with 2 units of *MspI (TLR2)*, *NcoI (TLR4)*, *LweI (IL6)*, *XagI (IL10)*, and restriction enzymes (New England Biolabs UK, Ltd., Hitchin, UK). Digestion was carried out at 37 °C for 3 h.

The enzymatic digestion was checked via the electrophoresis of 10 µL of the mixture on a 3% agarose gel containing a 0.5 mg/mL ethidium bromide solution. The gel was visualized on a UV transilluminator.

For *TLR2*-*Arg753Gln*, the Arg753 allele was characterized by the presence of the 104 and 25 bp fragments, while the Gln753 allele was characterized by the 129 bp fragment. For *TLR4*-*Asp299Gly*, the Asp299 allele was characterized by the presence of an undigested 188 bp fragment, while the Gly299 allele had two 168 and 20 bp fragments. For *IL*6-*174G/C*, the 174G allele was characterized by the presence of an undigested 610 bp fragment, while the 174C allele had two 367 and 243 bp fragments. For *IL10*-*1082G/A*, the 1082G allele was characterized by the presence of the 253, 97, and 27 bp fragments, while the 1082A allele was characterized by the presence of two 280 and 97 bp fragments.

**Figure 1:**
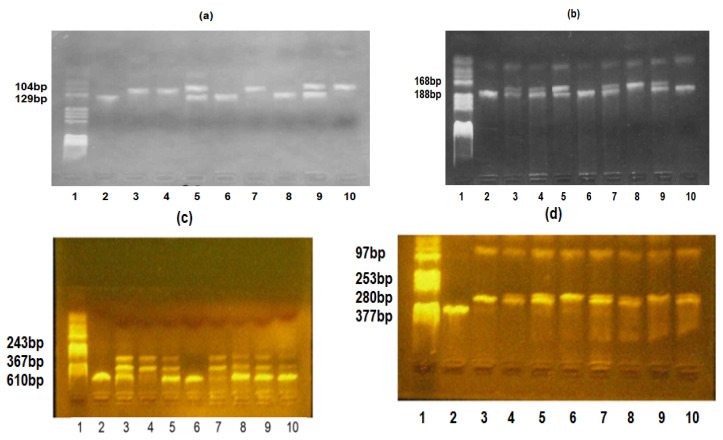
(**a**) *TLR2*-*Arg753Gln*, *TLR4*-*Asp299Gly*, *IL6*-*174G/C*, and *IL10*-*1082G/A* polymorphisms identification. *TLR2*-*Arg753Gln* polymorphism: lane 1—pBRHaeIIIDigest DNA molecular marker; lanes 2, 6, and 8—Gln/Gln genotype; lanes 3, 4, 7, and 10—Arg/Arg genotype; and lanes 5 and 9—Arg/Gln genotype. (**b**) *TLR4*-*Asp299Gly* polymorphism: lane 1—pBRHAeIII Digest DNA molecular marker, lanes 2, 6, and 10—Asp/Asp genotype, lanes 3, 4, 5, 7, and 9—Asp/Gly genotype, and lane 8—Gly/Gly genotype; (**c**) *IL6*-*174G/C* polymorphism: lane 1—pBRHaeIIIDigest DNA molecular marker, lanes 2 and 6—GG genotype, lanes 3, 5, 8, 9, and 10—GC genotype, and lanes 4 and 7—CC genotype. (**d**) *IL10 -1082G/A* polymorphism: lane 1—pBRHaeIIIDigest DNA molecular marker, lane 2—PCR fragment, lane 3—AA genotype, lanes 4, 5, 7, 8, and 10—GA genotype, and lanes 6 and 9—GG genotype.

Figure 1 shows the genotypes of the *TLR2*-*Arg753Gln*, *TLR4*-*Asp299Gly*, *IL6*-*174G/C*, and *IL10*-*1082G/A* polymorphisms.

The sequences of specific primers are presented in Table 1.

### 2.3. Statistical Analysis

Continuous quantitative variables measured in the sample of premature neonates were described using the arithmetic mean (standard deviation, SD) or median with interquartile interval IQR = (percentile 25th, 75th percentile), or count (%).

The differences in frequencies of qualitative characteristics between premature neonates with EOS and those without EOS were assessed using a Chi-squared test (χ^2^) or Fisher’s exact test. Student *t*-test with equal variances, Welch’s two-sample *t*-test, or the Mann–Whitney test was used to compare the distributions of quantitative characteristics between the studied independent groups.

Changes in the measured values of quantitative biochemical parameters between the initial assessment and the 7-day follow-up were evaluated using Student's *t*-test for dependent samples or Wilcoxon’s signed-rank test.

The associations between *TLR2*, *TLR4*, *IL6,* and *IL*10 gene polymorphisms and EOS susceptibility were tested using binomial logistic regression analysis, and the effect size of each tested association was estimated by odds ratio (OR) and a 95% confidence interval (95% CI). We also estimated the effects of studied gene polymorphisms conditional on gestational age group (very preterm versus moderate-late preterm [47].

The departure from Hardy–Weinberg equilibrium (HWE) for studied SNPs was performed on the non-EOS group using the Chi-square goodness-of-fit test, but when the genotype frequency was lower (<5), Fisher’s exact test was used.

The significance level (α) was set to 0.05. All statistical analyses were performed with R software, version 4.2.2 [48].

## 3. Results

### 3.1. Demographic, Anthropometric, and Clinical Characteristics of the Study Groups

A total of 36 preterm neonates were enrolled in the current study and were divided into two groups: clinEOS (n = 26, 72.22%) and Non-clinEOS (n = 10, 27.78%), according to the criteria previously mentioned. Demographic and clinical characteristics of the two groups are summarized in Table 2. Sex distribution did not differ significantly between the two groups (*p* = 0.836), whereas significant differences were observed in gestational age and birth weight (Table 2). Preterm neonates with clinEOS had a lower mean of gestational age [mean (SD): 29.4 (2.8) weeks vs. 32.6 (1.1); *p* = 0.00002] and birth weight [mean (SD): 1342.1 (446.5) g. vs. 1984 (376.9), *p* = 0.00031] compared to preterm neonates without clinEOS. Moreover, the preterm neonates with clinEOS had lower 1 min-Apgar scores than neonates without clinEOS [median (IQR): 6 (4 to 7) vs. 9 (7 to 9)]. We also noticed that the frequency distributions of maternal chorioamnionitis (*p* = 0.1547), urinary infection (*p* = 0.2853), delivery way (*p* > 0.05) and membrane rupture (*p* = 0.3169) were similar in the two groups.

### 3.2. Pointwise Comparison of Paraclinical Characteristics Between Preterm Neonates with Clinical EOS and Those Without Clinical EOS

No statistically significant differences in WBC, neutrophils, platelets, red blood cells, hemoglobin, and hematocrits were observed between the two groups on the first day of hospitalization (Table 3), except for C-reactive protein (*p* = 0.0099). We noticed that CRP values significantly increased in the EOS group compared with the non-EOS group (median, 95% CI: 0.80 [0.40, 1.15] vs. 0.30 [0.02, 0.50]). On the seventh hospital day, no differences between groups were noticed for any paraclinical parameters studied (*p* > 0.05).

The mean neutrophil count significantly decreased from baseline to day 7 of hospitalization in preterm neonates with clinical EOS (mean difference: 17.27%; 95% CI: [4.04%, 30.49%]; *p* = 0.0126) and those without clinical EOS (mean difference: 20.84%; 95% CI: [1.78%, 39.89%]; *p* = 0.0354). A significant decrease over time (baseline to day 7 of hospitalization) was noted for red blood cells (mean difference: 0.47; 95% CI: [0.28, 0.68]), hemoglobin (mean difference: 2.47; 95% CI: [1.66, 3.27]), and hematocrit (mean difference: 6.68; 95% CI: [4.43, 8.93]) only in premature neonates with clinical EOS (Table 3).

### 3.3. Associations of Studied SNPs’ Allele Frequencies with Odds of EOS in Premature Neonates

The genotype distributions of the four studied gene polymorphisms were in line with the Hardy–Weinberg equilibrium in the preterm neonates without clinical EOS except for IL10 (Table 4). The minor-allele frequency of the IL10-*1082G/A* gene polymorphism was higher in the clinEOS group than in the Non-clinEOS group (0.50 vs. 0.25), showing borderline statistical significance (*p* = 0.055). The other three SNPs showed no significant differences in allele frequencies between the groups (*p* > 0.05).

### 3.4. Associations Between Studied SNPs’ Genotype Frequencies and Odds of Clinical EOS in Premature Neonates

In the dominant model, the A/G + A/A variant genotype of the *IL10*-*1082G/A* polymorphism in the EOS group significantly increased the odds of EOS compared with the GG genotype (OR = 5.25, 95% CI: 1.07–25.70; *p* = 0.0322). After adjusting for gestational age group (<32 weeks vs. ≥32 weeks), the association between the *IL10*-*1082G/A* gene polymorphism and EOS did not reach statistical significance (adjusted OR = 5.72, *p* = 0.0752). There was no statistical evidence for a significant association of the *TLR4-Asp299Gly, TLR2-Arg753Gln*, and *IL6*-*174G* gene polymorphisms with odds of clinical EOS in premature neonates (*p* > 0.05; Table 5).

## 4. Discussion

Although there are some commonalities between pediatric and adult sepsis, there are important differences in pathophysiology, clinical presentation, and therapeutic approaches. The recognition and diagnosis of sepsis is a significant challenge in pediatric patients, as vital sign aberrations and examination findings are often subtle compared to those observed in adults [49].

The definition of sepsis in children is not yet complete; the application of Sepsis-3 to children has been attempted [50,51]. Therefore, the majority of studies used to establish evidence for these guidelines referred to the 2005 nomenclature in which severe sepsis was defined as (1) greater than or equal to two age-based systemic inflammatory response syndrome (SIRS) criteria, (2) confirmed or suspected invasive infection, and (3) cardiovascular dysfunction, acute respiratory distress syndrome (ARDS), or greater than or equal to two no cardiovascular organ system dysfunctions, and septic shock in children as severe infection leading to cardiovascular dysfunction (including hypotension, need for treatment with a vasoactive medication, or impaired perfusion) and “sepsis associated organ dysfunction” in children as severe infection leading to cardiovascular and/or non-cardiovascular organ dysfunction [5].

One of the most common infectious diseases developing in the neonatal care unit, neonatal sepsis, has a great impact on the survival rate in newborns.

The newborn has natural defense barriers, such as skin, mucous barriers, and vernix, that increase the defense barriers of the newborn against pathogens. Once these barriers are crossed, immunity must intervene. Recognition of the pathogen by local immune sentinel cells is the first step towards the development of an immune response [7].

Multiple classes of pathogen recognition receptors (PRRs) have been discovered that serve as detectors of pathogen-associated molecular patterns (PAMPs), including cell wall and membrane components, flagellum, nucleic acids, and carbohydrates [52]. A litany of PRR classes have been discovered, including the Toll-like receptors (TLRs), NOD-like receptors (NLRs), retinoic acid–inducible protein I-like receptors (RLRs), peptidoglycan recognition proteins, β2-integrins, and C-type lectin receptors [7].

In addition to its roles in leukocyte function (adhesion, phagocytosis, migration, and activation) and complement binding, complement receptor 3 (CR3, also known as MAC-1 and CD11b-CD18) functions as a pathogen sensor on the surface of phagocytes. Chemokine gradients produced by endothelial cells and local macrophages are necessary for effective and specific leukocyte attraction and accumulation. Damage-associated molecular patterns (or alarmins), such as intracellular proteins or mediators released by dying or damaged cells, may also activate PRRs [7]. If the pathogen is not contained locally and inflammatory homeostasis is not restored, SIRS may develop, leading to MODS and death [53].

Systemic inflammation is one of the causes of neonatal sepsis; the immune system of neonates, especially of the pre-term neonates, is not completely developed. So, managing this diagnosis is very difficult [54].

Early-onset sepsis (EOS) remains a serious and often fatal illness among infants born preterm, particularly among newborn infants of the lowest gestational age.

The standard diagnostic methods for EOS can generate false-negative results due to the manifestations of comorbidities associated with prematurity that overlap with the symptoms of sepsis. The clinical signs for EOS are from different systems and can be grouped as follows: (a) apnea, difficulty breathing, cyanosis; (b) tachycardia or bradycardia, poor perfusion or shock; (c) irritability, lethargy, hypotonia, seizures; (d) abdominal distension, vomiting, food intolerance, gastric residue, hepatomegaly; (e) unexplained jaundice; (f) body temperature instability; and (g) petechiae or purpura. To take into account the clinical signs, ideally, the newborn should show manifestations in three distinct systems, or two clinical signs in distinct systems associated with a maternal risk factor [13].

The current data support the hypothesis that TLRs behave differently in preterm newborns compared with at-term newborns. In at-term newborns, TLRs behave the same as in adults, while their expression is increased in preterm newborns. Differences in cytokine production between adult and neonatal innate mononuclear cells have been reported after the activation of TLRs’ bacterial ligands [55].

In our study, we found significant differences in C-reactive protein mean levels between the EOS and non-EOS groups. Wang X et al. [56] recommended a cut-off of 0.4 mg/dL for CRP within 4 h after birth among neonates born at <34 weeks for predicting EOS. In our study, the percentage of cases exceeding the threshold was 73.08% (19 cases), while 50% of the cases in the clinEOS group had a CRP value higher than 0.8 mg/dL.

The mean neutrophil count decreased in non-EOS premature neonates. In addition, mean neutrophils, red blood cells, hemoglobin, hematocrit, and C-reactive protein significantly decreased, and platelets significantly increased in the EOS preterm neonates during the study period.

Genetic variations located in the genes involved in inflammatory response could have an impact on the development, diagnosis, and outcome of neonatal sepsis. Confirming the mechanism of these gene variations will allow to develop new diagnostic tools, treatment strategies, and the chance to identify early patients at risk, improving the prognosis.

The current study aimed to observe if genotyping and specific SNP detection methods are associated with the early onset of neonatal sepsis in preterm neonates.

In 2015, Gao et al. performed a meta-analysis to demonstrate the association between *TLR2*-*Arg753Gln* polymorphism and the risk for sepsis and found a positive association regarding two genetic models, the allelic model and the dominant model. In the allelic model, the presence of the A allele is associated with a 1.76-fold (*p* = 0.03) increased risk for sepsis, and also, in a dominant model, carriers of the AA/GA genotypes had a 1.92-fold (*p* = 0.02) increased risk to develop sepsis [26].

According to Nachtigall et al., the presence of two SNPs of *TLR2*-*Arg753Gln* and *TLR4* -*Asp299Gly* was associated with a shorter time to onset of severe sepsis or septic shock in medical and surgical adult patients admitted to intensive care units [57]. Behairy et al. found a statistically significant association between the *TLR2*-*Arg753Gln* polymorphism and sepsis in the over-dominant variant G/G. The same study found an association between the *TLR4*-*Asp299Gly* polymorphism A/A-variant and infection with Acinetobacter baumannii (*p* = 0.001), non-specific Gram-negative bacilli infection, and sepsis in the adult population [58]. The distribution of the *TLR4*-*Asp299Gly* genotype in neonates suggests its association with an increased risk of culture-proven sepsis, according to Sljivancanin Jakovljevic et al. [59]. In the pediatric population, the *TLR4-Asp299Gly* polymorphism has been detected more frequently in patients with pyelonephritis, is more common in girls, and is always associated with *E. coli* [27,60]. In 2019, Attia et al. investigated 36 bacteremia cases with early or late onset neonatal sepsis for the presence of *TLR2*-*Arg753Gln*, but they concluded that there is no association of this polymorphism with the risk of neonatal sepsis [61]. Khaled et al. in 2020 [62] analyzed 30 neonates with confirmed sepsis for the presence of *TLR2* and *TLR4* polymorphisms and found a different and significant distribution of these in the sepsis group as compared with controls (*p* = 0.016). They found longer duration therapy in cases positive for these polymorphisms as compared with negative cases, suggesting a role of *TLR2* and *TLR4* polymorphism in innate immune response [62]. Sampath et al. (2013) and Lorenz et al. (2000) demonstrated that *TLR2*-*Arg753Gln* and *TLR4*-*Asp299Gly* polymorphisms are associated with Gram-positive and Gram-negative sepsis, respectively [63,64].

In the present study, a significant difference in the frequency distributions of the *TLR4*-*Asp299Gly* gene polymorphism between the clinEOS and nonclin-EOS preterm newborn groups was not found. Regarding the *TLR2*-*Arg753Gln* polymorphism, the minor allele *Gln753* frequency was higher in the clinEOS group than in the nonclin-EOS group (32.69% vs. 25.00%). Even though the risk of developing clinEOS increased in the presence of this allele in the sample studied, the results were not statistically significant. Furthermore, the analysis of different genotypic testing models revealed an increased estimated risk of developing clinEOS in the presence of *TLR2*-*Arg753Gln* variant genotypes (co-dominant model-OR 1.38, dominant model-OR 1.5, recessive model-OR 1.64). But the results failed to show statistical significance for the association in both univariate logistic regression analysis and multivariate analysis after adjusting for gestational age.

*TLR2-Arg753Gln* polymorphism is located in the intracellular Toll/interleukin 1 receptor domain of the *TLR2* gene and impairs tyrosine phosphorylation, dimerization with TLR6, and MyD88 recruitment with an effect on nuclear factor κB activation. These events determine decreased cytokines secretion, increased susceptibility to bacterial diseases, and sepsis [28,64].

Immune cells such as monocytes and macrophages synthesized cytokines. Some of these have an inflammatory role. There are pro-inflammatory cytokines, such as IL6, and also anti-inflammatory cytokines, such as Il10. Pro-inflammatory cytokines can promote the inflammatory response, and anti-inflammatory cytokines suppress excessive inflammatory responses. Both have a role in systemic inflammation and sepsis [65].

IL6 is a vital pro-inflammatory cytokine generated by various cells such as leukocytes, endothelial cells, myocytes, adipocytes, and fibroblasts [32,66]. Several polymorphisms in the *IL6* gene’s promoter region have been identified. Among these, the common polymorphism-*174G/C* (rs1800795) contains a DNA-binding site for the nuclear factor IL6, a transcription factor that can interact with estradiol receptor complexes to regulate the expression of the *IL6* gene [32,67].

The results regarding the association between *IL6*-*174G/C* polymorphism and the risk for early-onset neonatal sepsis are controversial. In various genetic studies, IL6 polymorphisms in the promoter region have been shown to increase the risk of sepsis, although with inconsistent results. Zhao et al. (2022) performed a study on full-term neonates (gestational age ≥ 37 weeks and <40 weeks) and found almost the same frequency of CC (12% vs. 15%) and CG (23% vs. 33%) genotypes in sepsis and control neonates (*p* = 0.008) [68]. But there was a higher frequency of the heterozygous carriers in critically ill as compared with non-critically ill groups, based on the Neonatal Critical Illness Score (NCIS) [69]. A meta-analysis of 16 studies performed by Hu P. et al. that systematically examined the correlation of IL6 gene polymorphisms (-*174G/C* and -*572C/G*) with susceptibility to sepsis found that the *IL6*-174G/C gene polymorphism was not statistically associated with the risk of sepsis in adults, neonates, or the pediatric population [32]. On the other hand, Mao et al. (2017) demonstrate a higher frequency of the CC174 genotype in patients with sepsis (OR 4.45, *p* < 0.01) and an increased odds for pneumonia-induced sepsis in carriers of the IL6-174C allele [70]. What is interesting, Allam et al. (2015) found that the *IL6* G174 allele was associated with early-onset neonatal sepsis in Saudi Arabia [71]. Mostafa et al., in 2022, investigated patients with different stages of sepsis and found an association between *IL6 G174C p*olymorphism and the severity of sepsis [72]. Ferdosian et al., in 2021, performed a meta-analysis and demonstrated no risk of sepsis in children positive for *IL6*-*174G/C*, but when they were divided for subgroup analysis, they found a higher risk in Caucasians and Africans [73]. The meta-analysis performed by Liang et al. (2024) suggest 1.471-fold increased risk to develop neonatal sepsis in carriers of the CC genotype for *IL6*-*174G/C* [74]. Although the groups of preterm neonates studied had different demographic characteristics, our study did not find statistically significant results between the presence of *IL6* SNPs and the risk of developing clinEOS in premature neonates. The results are in line with those obtained by Zhao et al. (2022) and Varljen et al. (2019) [68,75].

IL10 is an anti-inflammatory cytokine. The key producers of IL10 in systemic inflammation are resident macrophages of the reticuloendothelial system. It also has an immunosuppressive role in the body. The *IL10* gene is located on chromosome 1 and has five exons. There are some studies which investigated one polymorphism in the *IL10* gene, a guanine substitution with adenine in nucleotide 1082 of the gene (*IL10*-*1082G/A*) as a factor influencing IL10 levels and a potential candidate for the development of sepsis, both in adults and newborns. Studies have investigated the *IL10*-*1082G/A* polymorphism concerning the susceptibility to sepsis in the Asian population and Afro-Colombian patients [76]. A meta-analysis of 11 studies on the adult population concluded that the *IL10*-*1082G/A* polymorphism is significantly associated with susceptibility to sepsis in Asian populations [40]. At the same time, the AA genotype of the *IL10*-*1082G/A* polymorphism is a risk factor for elevated IL10 production and the development of sepsis due to Gram-negative bacteria, especially in Afro-Colombian patients [4]. On the other hand, Mao et al. (2017) [70] confirmed a higher frequency of the AA genotype for the *IL10*-1082G/A polymorphism in patients with sepsis (57.98%) and also in pneumonia-induced sepsis (45.88%) groups as compared with healthy subjects (38%). The results suggest that the *IL10*-A1082 allele could be an indicator for pneumonia-induced sepsis [70]. This polymorphism could affect IL-10 transcription. Treszl et al. (2003) demonstrate that *IL10*-*1082G/A* polymorphism has no role in neonatal sepsis [77]. On the contrary, Abu-Maziad et al. (2010) investigated infants with low birth weight and also preterm infants and found a reduced risk of sepsis in carriers of the GG genotype for *IL10*-*1082G/A* polymorphism [78]. Performing a meta-analysis in 2024, Liang et al. analyzed different genetic models and obtained the following results. In a dominant model, carriers of the AG + AA genotype had 1.731 (*p* = 0.016) increased odds of neonatal sepsis. In the recessive model, newborns with the AA genotype had a 1.702 (*p* = 0.002) increased risk to develop neonatal sepsis. Also, in a homozygous gene model the risk increased to 2.282 (*p* = 0.002) in carriers of the AA genotype [74].

In the present study, the results suggest that carriers of the *IL10*-1082A allele had a 2.91-fold increased risk of developing clinEOS, with marginal significance (*p* = 0.055). The results of the univariate logistic regression analysis revealed that the IL10-*1082G/A* polymorphism increased the odds of clinEOS in the dominant model (*p* = 0.032). After controlling for gestational age group, the *IL10*-1082G/A gene polymorphism remained a risk factor for clinEOS, with a tendency toward statistical significance. Our results suggest the role of *IL10*-*1082G/A* polymorphism as a possible predictive risk factor for early neonate sepsis. Even if our results were obtained in neonates with sepsis, we could observe that these are in agreement with those obtained by Pan et al. (2015), who investigated adult sepsis [79]. The results of our study are also in agreement with the results published by Liang et al. (2024) in a recent meta-analysis involving 3338 neonates with sepsis [74].

One of the major roles in sepsis is played by the inflammatory response to infection (de Pablo et al., 2014) [80]. Rees et al. (2002) demonstrate that IL6 and IL10, as pro-inflammatory cytokines, represent components mediating sepsis [81]. The levels of IL6 increase as a first responder to the innate cell immune response to infection. The *IL6*-*174G/C* polymorphism influences the IL6 levels and also the transcription of the protein, having a role in septic shock [72]. In the meantime, IL10 is related to down regulation of the inflammatory mediator production in sepsis [82].

The strong points of the current study consist of the fact that:

(i) It is an innovative study that directs the spotlight onto the effect of the presence of *TLR2*, *TLR4*, *IL6*, and *IL10* polymorphisms in the context of early neonatal sepsis in preterm neonates. There are studies in the literature on the involvement of SNPs in sepsis, but not on groups of premature newborns.

(ii) The interesting conclusion is that SNP *IL10*-*1082G/A* increases predisposition to sepsis.

Our study has several limitations: (i) the use of a non-probabilistic sampling method (a convenience sample of premature) might alter the representativeness of the sample; (ii) most of the cases were diagnosed on clinical manifestation due to the small number of blood cultures; (iii) the small number of clinEOS cases and Non-clinEOS premature neonates included in the SNP analysis might influence the stability of the estimated regression coefficients; (iv) the lack of significant statistically results regarding the association between TLR4 and IL6 SNPs and the odds of developing clinEOS in premature neonates may be partly explained by limited statistical power due to the small number of premature neonates or maybe the studied gene polymorphisms exert only a modest biological effect that which could not be generalized in pediatric population of premature neonates; (v) the adjustment for prenatal, perinatal, and postnatal maternal factors of EOS was not performed in the current study due to the small number of neonates, (vi) the clinical characteristics of studied sample are unbalanced and (vii) the results with marginal significance should be interpreted with caution (we performed a post hoc power calculation using “genpwr” R package version 1.0.4 [60] and after considering a sample size n = 36, MAF = 0.4 (mean), proportion of cases in the sample = 0.722, OR = 5, and significance level = 0.05, the study power was estimated at 54.89%) and (viii) given that the association between *IL10*-*1082G/A* gene polymorphisms and clinical EOS susceptibility lost significance after adjustment for gestational age group (very preterm versus moderate-late preterm), future studies should test whether gestational age group mediates the relationship between IL10-*1082G/A* gene polymorphisms and clinical EOS.

## 5. Conclusions

Based on our observations, the genotypic testing models revealed that in the codominant model, the *IL10*-*1082G/A* polymorphism increased the odds of early-onset sepsis in premature neonates. Even though there was a trend toward statistical significance regarding the association of the *IL10*-*1082G/A* gene polymorphism with the odds of EOS in the allelic model, future studies should confirm the impact of individual alleles on early-onset sepsis in premature neonates.

## Figures and Tables

**Table 1:** Specific primers were used to identify TLR2-*Arg753Gln*, TLR4-*Asp299Gly*, IL6-*174G/C*, and IL10-*10882A/G* polymorphisms.

Genetic Variation	Primers’ Sequences
***TLR2-Arg753Gln***	FW: 5′-CAT TCC CCA GCG CTT CTG CAA GCT CC-3′
	RV: 5′-GGA ACC TAG GAC TTT ATC GCA GCT C-3′
***TLR4-Asp299Gly***	FW: 5′-GAT TAG CAT ACT TAG ACT ACT ACC TCC ATG-3′
	RV: 5′-GAT CAA CTT CTG AAA AAG CAT TCC CAC-3′
***IL6-174G/C***	FW: 5′-CAG AAG AAC TCA GAT GAC TGG-3′
	RV: 5′-GCT GGG CTC CTG GAG GGG-3′
***IL10-1082G/A***	FW: 5′-CCA AGA CAA CAC TAC TAA GGC TCC TTT-3′
	RV: 5′-GCT TCT TAT ATG CTA GTC AGG TA-3′

FW—forward; RV—reverse.

**Table 2:** Demographic and clinical characteristics of preterm neonates stratified by the presence of clinical early-onset sepsis.

Postnatal Characteristics	Non-clinEOS Group (*n*= 10)	ClinEOS Group (*n* = 26)	*p*-Value
Male Gender, *n* (%)	5 (50.0)	12 (46.2)	0.8360 ^a^
Gestational age (weeks), mean (SD)	32.6 (1.1)	29.4 (2.8)	0.00002 *^,b^
Delivery way, *n* (%)			1.000 ^a^
Vaginal	7 (70.0)	19 (73.1)	
Cesarean	3 (30.0)	7 (26.9)	
Birth weight (gram), mean (SD)	1984 (376.9)	1342.1 (446.5)	0.00031 *^,b^
Neonate length (cm), mean (SD)	43.3 (2.9)	37.4 (6.4)	0.0005 *^,b^
Head circumference (cm), mean (SD)	30.2 (1.8)	26.5 (2.7)	0.0004 *^,b^
Apgar 1 min, median (IQR)	9 [7, 9]	6 [4, 7]	0.00009 *^,c^
Apgar 5 min, median (IQR)	9 [8, 9]	8 [7, 8]	0.00004 *^,c^

n = absolute frequency; SD = standard deviation; IQR = [percentile 25th, percentile 75th]; ^a^ Chi-square test; ^b^ a two-sided Student’s *t*-test for two independent samples; ^c^ exact *p*-values obtained from Mann–Whitney test; * significant result: *p* < 0.05.

**Table 3:** Comparisons of laboratory characteristics between premature neonates with clinEOS vs. Non-clinEOS.

Variables	Time Points	Non-clinEOS Group (*n*= 10)	ClinEOS Group (*n* = 26)	Within Group *p*-Value	Between Group *p*-Value	Between Group Adjusted *p*-Value
WBC (mm^3^), median (IQR)	T0	12,785 [10,140, 15,292.5]	11,985 [8145, 16,090]	–	0.6894	–
	T1	10,455 [8742.5, 11,475.0]	12,285 [9026.3, 16,942.5]	Non-clinEOS: 0.1309 clinEOS: 0.5009	0.2411	0.9233
Neutrophils (%), mean (SD)	T0	53.6 (21.6)	56.8 (23.8)	–	0.7068	–
	T1	32.7 (13.4)	39.6 (16.9)	Non-clinEOS: 0.0354 * clinEOS: 0.0126 *	0.2588	0.2180
Platelets (mm^3^), mean (SD)	T0	280.2 (46.2)	236.5 (78.2)	–	0.1077	–
	T1	366.5 (70.9)	345.6 (155.8)	Non-clinEOS: 0.0031 * clinEOS: 0.0004 *	0.5855	0.7025
Red blood cells: 10 × 12/L, mean (SD)	T0	4.36 (0.44)	4.33 (0.35)	–	0.8422	–
	T1	4.12 (0.56)	3.86 (0.48)	Non-clinEOS: 0.0524 clinEOS: 0.00007 *	0.1834	0.1735
Hemoglobin (g/L), mean (SD)	T0	16.42 (1.94)	16.25 (1.50)	–	0.7927	–
	T1	15.14 (2.38)	13.79 (1.93)	Non-clinEOS: 0.0227 * clinEOS: 0.000001 *	0.0862	0.0719
Hematocrit (%), mean (SD)	T0	48.40 (6.39)	47.98 (4.54)	–	0.8280	–
	T1	44.92 (7.36)	41.31 (5.70)	Non-clinEOS: 0.0488 * clinEOS: 0.000002 *	0.1261	0.0960
C-reactive protein (mg/dL), median (IQR)	T0	0.30 [0.09, 0.4]	0.80 [0.3, 1.2]	–	0.0099 *	–
	T1	0.09 [0.08, 0.11]	0.14 [0.06, 0.38]	Non-clinEOS: 0.058 clinEOS: 0.0435 *	0.1417	0.7959

T0: baseline; T1: at 7 day of hospitalization; SD = standard deviation; IQR = [percentile 25th, percentile 75th]; within group *p*-values were obtained from Wilcoxon test or paired Student *t*-test; between group *p*-values were obtained from Wilcoxon rank sum test or Student *t*-test for independent samples; between group adjusted *p*-values were obtained from ANCOVA controlling for baseline values or non-parametric ANCOVA; * significant result: *p*-value < 0.05.

**Table 4:** Frequency distributions of *TLR4*, *TLR2*, *IL6*, and *IL10* alleles and their associations with the presence of clinical EOS.

SNPs	Minor Allele	Relative Frequencies (%)		HWE *p*-Value ^a^	OR, [95% CI]	*p*-Value
		**clinEOS** **Group**	**Non-ClinEOS** **Group**			
*TLR4-Asp299Gly*	Gly	15.38	20	0.3065	0.72 [0.19, 3.12]	0.6379
*TLR2-Arg753Gln*	Gln	32.69	25	0.4799	1.46 [0.46, 5.11]	0.5257
*IL6-174G/C*	C	32.69	50	0.1998	0.49 [0.17, 1.43]	0.1742
*IL10-1082G/A*	A	50	25	0.0464	2.91 [0.96, 10.28]	0.0550

SNPs: single-nucleotide polymorphisms; HWE: Hardy–Weinberg equilibrium; ^a^ calculated in the Non-clinEOS group; OR: unadjusted odds ratio; 95% CI: 95% confidence interval.

**Table 5:** Associations between SNPs and clinical EOS: results of genotypic model analysis.

SNP	Model of Inheritance	Genotype	Non-clinEOS Group *n (%)*	clinEOS Group *n (%)*	OR (95% CI)	*p*-Value	Adjusted OR ^a^ (95% CI)	*p*-Value
*TLR4* *Asp299Gly*	Co-dominant	Asp/Asp	7 (70.0)	20 (76.9)	Reference	0.9138	Reference	0.8520
		Asp/Gly	2 (20.0)	4 (15.4)	0.70 [0.10, 4.69]		0.84 [0.08, 9.19]	
		Gly/Gly	1 (10.0)	2 (7.7)	0.70 [0.05, 8.97]		0.36 [0.01, 12.74]	
	Dominant	Asp/Asp	7 (70.0)	20 (76.9)	Reference	0.6712	Reference	
		Asp/Gly + Gly/Gly	3 (30.0)	6 (23.1)	0.70 [0.14, 3.58]		0.66 [0.08, 5.42]	0.6991
	Recessive	Asp/Asp − Asp/Gly	9 (90.0)	24 (92.3)	Reference	0.8254	Reference	0.5837
		Gly/Gly	1 (10.0)	2 (7.7)	0.75 [0.06, 9.32]		0.37 [0.01, 12.81]	
*TLR2* *Arg753Gln*	Co-dominant	Arg/Arg	6 (60.0)	13 (50.0)	Reference	0.8430	Reference	0.8885
		Arg/Gln	3 (30.0)	9 (34.6)	1.38 [0.27, 7.04]		0.89 [0.11, 7.31]	
		Gln/Gln	1 (10.0)	4 (15.4)	1.85 [0.17, 20.26]		1.86 [0.11, 32.41]	
	Dominant	Arg/Arg	6 (60.0)	13 (50.0)	Reference	0.5892	Reference	0.9062
		Arg/Gln + Gln/Gln	4 (40.0)	13 (50.0)	1.50 [0.34, 6.59]		1.12 [0.17, 7.22]	
	Recessive	Arg/Arg + Arg/Gln	9 (90.0)	22 (84.6)	Reference	0.6668	Reference	0.6363
		Gln/Gln	1 (10.0)	4 (15.4)	1.64 [0.16, 16.73]		1.93 [0.12, 30.93]	
*IL6* *174G/C*	Co-dominant	G/G	1 (10.0)	9 (34.6)	Reference	0.0896	Reference	0.4715
		G/C	8 (80.0)	17(66.4)	0.24 [0.03, 2.20]		0.43 [0.03, 5.73]	
		C/C	1 (10.0)	0 (0.0)	ND		ND	
	Dominant	G/G	1 (10.0)	9 (34.6)	Reference	0.1140	Reference	0.4643
		G/C + C/C	9 (90.0)	17 (65.4)	0.21 [0.02, 1.93]		0.39 [0.03, 5.25]	
	Recessive	G/G + G/C	9 (90.0)	26 (100.0)	Reference	0.2778	Reference	0.3012
		C/C	1 (10.0)	0 (0.0)	ND		ND	
*IL10* *1082G/A*	Co-dominant	G/G	7 (70.0)	8 (30.8)	Reference	0.0779	Reference	0.1579
		A/G	1 (10.0)	10(38.5)	8.75 [0.88, 86.57]		2.38 [0.11, 50.05]	
		A/A	2 (20.0)	8 (30.8)	3.50 [0.55, 22.30]		8.35 [0.81, 85.85]	
	Dominant	G/G	7 (70.0)	8 (30.8)	Reference	0.0322 ^*^	Reference	0.0752
		A/G + A/A	3 (30.0)	18 (69.2)	5.25 [1.07, 25.70]		5.72 [0.76, 43.31]	
	Recessive	G/G + A/G	8 (80.0)	18 (69.2)	Reference	0.5091	Reference	0.0659
		A/A	2 (20.0)	8 (30.8)	1.78 [0.31, 10.32]		7.33 [0.75, 71.29]	

SNPs: single-nucleotide polymorphisms; ND: not determined; OR: unadjusted odds ratio; ^a^ adjusted for gestational age groups (<32 weeks vs. ≥32 weeks); 95% CI: 95% confidence interval; *p*-values estimated from unconditional logistic regression (Wald test); * significant result: *p*-value < 0.05.

## Data Availability

The raw data involved in this study can be obtained upon reasonable request addressed to Lucia M. Procopciuc (lprocopciuc@umfcluj.ro) and Melinda Baizat (melindabaizat@gmail.com).
